# The Teeth of Ancient Hawaiians

**Published:** 1894-03

**Authors:** J. M. Whitney


ARTICLE VIII.

### THE TEETH OF ANCIENT HAWAIIANS.

BY DR. J. M. WHITNEY.

We have been taught that primitive peoples, living in simple conditions, were in a great measure free from dental caries as we see it in the mouths of our patients, and that many forms of dental diseases with which we have to contend were, with them, wholly unknown. This seems to me an erroneous teaching, from what can be learned from these records. An exceptional opportunity of becoming acquainted with the crania of the ancient people of these islands during the twenty-four years of my residence here has convinced me that both in the case of those buried in the caves, and of those more recently found in the sand, not more than 25 per cent. have been free from caries, irregularity, or disease. Indeed, I think I have discovered every form of dental disease known to our practice. Dental caries in all its many types, necrosis of the teeth, erosion, alveolar abscess, pyorrhea alveolaris, disease of the antrum of highmore, necrosis of the maxillary, ankylosis of the jaw, salivary calculus, etc.

Here was a well-developed osseous system; the individual was trained to exercise of the kind that would develop every part of the structure. Living on an abundance of the simplest, yet the most nutritious and bone-developing foods that would not cling to the teeth, but would exercise and clean them with not an element lacking required by our present knowledge, yet the same dental disease from which we suffer, burdened the lives of the ancient Hawaiians.

While this is true, I have been interested to find that the teeth of those who died before civilization had introduced to the people peculiar constitutional diseases, acid fruits and vegetables, fine flour, and various foods, were much less seriously attacked by disease than afterwards. As a general statement the teeth would be found clean, and when caries existed it was here and there in teeth of both maxillaries and on both sides, but not so pervading as found in the more recent crania, or in the mouths especially of the young of the present time.

We have often accounted for the irregularity of teeth found so common among Americans, by the mixture of races of which our nation is composed. We say that the wide teeth of the large jaw of one race, being crowded into the narrow jaw of another race with which it has mingled, would of neccssity produce an irregular arch. But here is a people, isolated from all others for at least thirteen hundred years, with no admixtures of races; yet irregularity of the teeth of both maxillaries was almost as common as it is among the mixed races of to-day. It would be difficult to give a good reason why a fixed type for the mouth of this race should not have existed a thousand years ago, and that all, with rare exceptions, should have been modeled from it, had nature designed that there should be absolute uniformity in her work.

Among the crania I have examined I have noticed that the teeth are set closely together and well rounded, and that the dense part of the enamel, near the cutting-edge or grinding-surface, strikes its fellow at that point, the whole being held firmly by the buttressed third molar. This type is somewhat fixed.

Perhaps next to dental caries, the greatest source of oral disorders among these people was the irregularity of the third molar, often producing in them as serious consequences as with us; while its failure to erupt was nearly or quite as common as we find it in our daily practice, so that we cannot argue, from these remains at least, that the coming man is to be deprived of this useful organ.

The relation of food and disease to the health of the dental organs is strongly demonstrated from a study of the changes shown in the teeth of those buried in the oldest caves, and so down through the more recent burials in the sand. Then of those who were the old people a quarter of a century ago, whose childhood was passed before civilization had touched their primitive life and their grandchildren who are now in our schools. These children, as shown by actual examination, have but little better teeth than their white school-fellows. Their parents, however, may have better teeth than the children, but it would be an exception if they had not been to the government physician and had aching teeth removed, while the grandparents, the old men and women whom I found when I first went to the islands, had teeth proximating those found in the old caves, though not as good.—*Items of Interest.*

